# Lithium sulfonate-grafted poly(vinylidenefluoride-hexafluoro propylene) ionomer as binder for lithium-ion batteries

**DOI:** 10.1039/c8ra02122h

**Published:** 2018-05-31

**Authors:** Zhiqun Wang, Shaokang Tian, Shangda Li, Lei Li, Yimei Yin, Zifeng Ma

**Affiliations:** School of Chemistry and Chemical Engineering, Shanghai Key Lab of Electrical Insulation and Thermal Aging, Shanghai Jiao Tong University Shanghai 200240 China lilei0323@sjtu.edu.cn; Shanghai Electrochemical Energy Devices Research Center, School of Chemistry and Chemical Engineering, Shanghai Jiaotong University Shanghai 200240 China

## Abstract

Lithium sulfonate-grafted poly(vinylidenefluoride-hexafluoro propylene) P(VDF-HFP) ionomers are synthesized through covalent attachment of taurine and used as binder for the LiFePO_4_ cathode of lithium-ion batteries(LIBs). The incorporation of the ionomer binders will add ionic conducting channels inside the electrodes, and prevent electrolyte depletion during rapid charge–discharge processes. It leads to an improved performance of LIBs using the ionomer binders including cycling stability and rate capability compared to that of LIBs using non-ionic binders (PVDF and PVDF-HFP). Therefore, the lithium sulfonate-grafted P(VDF-HFP) ionomers offer a new route to develop high-power LIBs.

## Abstract 2

Lithium sulfonate-grafted PVDF-HFP was successfully synthesized and used as binder for lithium ion batteries, improving electrochemical performance.

## Graphical abstract

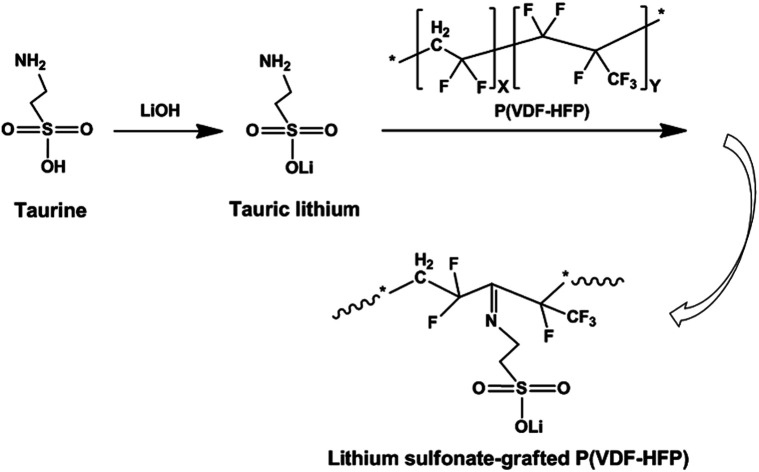

## Introduction

Rechargeable lithium-ion batteries (LIBs) have attracted considerable attention as energy storage devices for electric vehicles and portable electronic devices due to their high energy density, portability and high voltage.^1–4^ These electric vehicles and electronic devices require batteries with high rate capability. Typical LIB electrodes are obtained by mixing electroactive materials, carbon black and binder. There is at least 2 wt% to 10 wt% of binder within the electrodes. Poly(vinylidene fluoride) (PVDF) is commonly used as electrode binder and polymer electrolytes^5,6^ for LIBs due to its good electrochemcial stability, binding capability, and high adhesion to the electrode materials and current collectors.^7–12^ However, PVDF non-ionic polymer is an insulator, which does not have any electronic or ionic functionality. Therefore, the electrode using PVDF as binder will lead to high polarization resistance at high rate capability, finally resulting in a poor rate capability of LIBs.^13–16^ Researchers have developed some new binders such as polyrotaxanes, cross-linked carboxymethyl cellulose and citric acid polymer, polyacrylic acid and poly(ethylene glycol) diglycidyl ether with polyethylenimine for LIBs with silicon anode,^17–25^ lithium–sulfur batteries^26–32^ and other rechargeable batteries.^33–39^

The binders with ionic conductivity are of great interest, due to the fact that they will enhance lithium ion conductivity with high rate capability of LIBs. Recent studies have shown that using lithiated ionomers as binders will provide a path between electroactive materials and electrolytes, which reduces the polarization resistance and increases the ionic conductivity within the electrodes. Li *et al.* used polyacrylic acid (PAA) as binder to lower the polarization resistance of the LiFePO_4_ cathode.^40^ By the addition of lithiated perfluorinated sulfonic ionomers such as Nafion^41,42^ and Dow^43^ to the lithium cathode, cycling stability at large current density of LIBs was improved. Shi *et al.* synthesized lithiated poly(perfluoroalkyl sulfonyl)imde (PFSILi) ionene and blended it with PVDF to make it as ionic binder for the LiFePO_4_/C cathode.^44^ The cathode with this ionic composite binder exhibited a higher rate capacity compared with the PVDF non-ionic binder. Chui *et al.* adopted lithiated perfluorosulfonate ionomer as the binder for LiMn_2_O_4_ cathodes.^41^ Wei *et al.* prepared the LiFePO_4_/C cathode with sulfonated polyether ether ketone with pendant lithiated fluorinated sulfonic groups (SPEEK-FSA-Li) as binder to reduce the Li^+^ concentration polarization and electrolyte depletion during rapid charge–discharge processes.^45^

Since poly(vinylidenefluoride-hexafluoro propylene) (P(VDF-HFP)) polymer has higher amorphousity and lower glass transition temperature (*T*_g_), Hu *et al.* reported that the LiFePO_4_ cathode using P(VDF-HFP) as binder showed better electrochemical performance including cycling stability and rate capability compared to the LiFePO_4_ cathode using PVDF as binder.^46^ However, P(VDF-HFP) polymer still does not have intrinsic ionic functionality, so that it is difficult to improve rate performance of LIBs.

**Scheme 1:**
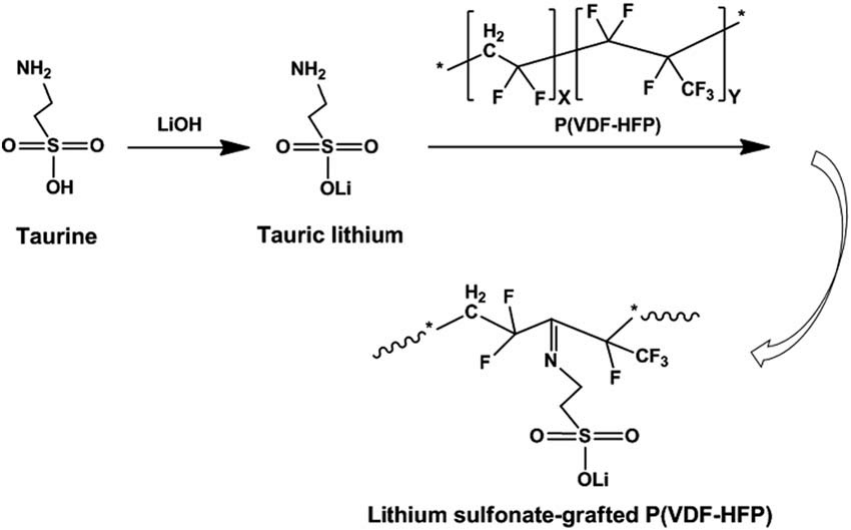
Schematic illustration of synthesis route of lithium sulfonated grafted P(VDF-HFP).

Herein, we synthesized lithium sulfonate-grafted P(VDF-HFP) ionomers with different content of Li^+^ and used them as binders for the LiFePO_4_ cathode of LIBs. It is well known that amine is easily grafted onto fluoropolymer main chains as functional groups owing to the strong polarity of C–F bonds.^47,48^ In our experiments, taurine, one of sulfur-containing amino acids, was directly grafted onto P(VDF-HFP) polymer chains through one reaction in solution (see Scheme 1). The content of Li^+^ of the lithium sulfonate-grafted P(VDF-HFP) ionomers was controlled by various taurine feed contents. Electrochemical performance of these ionomers as binders for the LiFePO_4_ cathodes of LIBs were investigated by electrochemical impedance spectroscopy, charge–discharge testing, cycling voltammetry and 180° peel testing. The performance of cathodes including cycling stability, rate capability and adhesion strength were improved compared to the cathodes using P(VDF-HFP) as binders. To the best of our knowledge, it is the first time to report that the lithium sulfonate-grafted P(VDF-HFP) ionomers were used as binders in LIBs.

## Experimental

### Synthesis lithium sulfonate-grafted P(VDF-HFP)

First, 4.0 g taurine and 0.768 g LiOH (1 : 1 mole ratio) were dissolved in 30 mL of deionized water, and stirred at 40 °C for 5 h. After that, the solution was free-dried for 48 h to obtain tauric lithium. Second, 2.0 g P(VDF-HFP) (*M*_w_ = 115 000, *X* = 9, *Y* = 1 Solvay Solexis Inc.) polymer was added into 38.0 g dimethylacetamide (DMAC) solvent with magnetic stirring for 2 h. Then desired amounts of tauric lithium and MgO (2 : 1 mole ratio) were added into the solution. In our experiments, the weight ratios of tauric lithium and P(VDF-HFP) were kept at 5 wt%, 15 wt% and 20 wt%, respectively. The mixture was heated and kept at 100 °C for 10 h in argon atmosphere, and then cooled down to room temperature. After that, the product was precipitated from diethyl ether and washed by deionized water till the pH = 7. Finally, the lithium sulfonate-grafted P(VDF-HFP) ionomer was dried at 60 °C under vacuum overnight to remove the residual solvent. The content of Li^+^ of the lithium sulfonate-grafted P(VDF-HFP) ionomers was determined by titration: the ionomers were kept in 1 M HCl aqueous solution for 72 h to substitute Li^+^ by H^+^ of ionomers, then soaked in 3.4 M NaCl aqueous solution for 30 h and finally back titrated with 0.5 M NaOH using phenolphthalein as an indicator. The ionomers with the taurine feed content 5 wt%, 15 wt% and 20 wt% were denoted as grafted-P(VDF-HFP)-5, grafted-P(VDF-HFP)-15 and grafted-P(VDF-HFP)-20, respectively.

### Characterization

The chemical structure of the lithium sulfonate-grafted P(VDF-HFP) was analysed by Fourier transform infrared spectroscopy (ATR-FTIR, Spectrum 100, Perkin Elmer, Inc., USA) with a wavenumber range of 4000–400 cm^−1^, and Nuclear magnetic resonance (^1^H NMR) spectra (BioSpin Corp., Germany) using a 400 MHz spectrometer (AVANCE III HD 400 MHz Bruker) in DMSO-d_6_ instrument at 25 °C. X-Ray Photo Electron Spectroscopy (XPS) was performed using an X-ray photoelectron spectrometer (AXIS ULTRA DLD, Kratos Analytical Ltd., UK) with a monochromatic Al Kα source (1486.6 eV).

### Electrochemical measurements

The working electrode of the lithium-ion battery was prepared by mixing LiFePO_4_ (Pylon Technologies Co., Ltd., China), Super P (Timcal Graphite & Carbon, Switzerland) and the lithium sulfonate-grafted P(VDF-HFP) ionomers at a weight ratio of 80 : 10 : 10 in *N*-methylpyrrolidone (NMP) solvent. The mixed slurry was coated on aluminium foil and dried under vacuum at 80 °C overnight. For comparison, the LiFePO_4_ cathode with P(VDF-HFP) (*M*_w_ = 115 000, Solvay Solexis Inc.) binders was prepared in the same way. The CR2025-type half-cells were assembled with lithium metal, Celgard 2400 separator, electrolyte (1 M LiPF_6_ in a 1 : 1 (wt : wt) EC/DMC) and the prepared working electrode. The test cells were assembled in an argon-filled glove box. Galvanostatic cycling was performed on Land CT2001A tester (Wuhan, China) between 2.4 and 4.3 V (Li *vs.* Li^+^). Electrochemical impedance spectroscopy (EIS) was accomplished with an Autolab frequency response analyzer from a frequency range of 0.01 Hz to 100 KHz. Cyclic voltammetry (CV) was measured between 2.4–4.3 V at a scanning rate of 0.2 mV s^−1^ by Autolab PGSTAT302 electro-chemical test system (Eco Chemie, the Netherlands) at room temperature.

### Adhesion characterization

The adhesion strength of the binders between the coating of the LiFePO_4_ electrode and the Al current collector were measured by a 180 peeling test using an omnipotent electronic stress–strain tester (MTS, Criterion 43). The Al currents coated electrodes were cut to a strip of 9 mm width. The strip was pulled at a speed of 1.0 cm min^−1^.

## Results and discussion

**Scheme 2:**
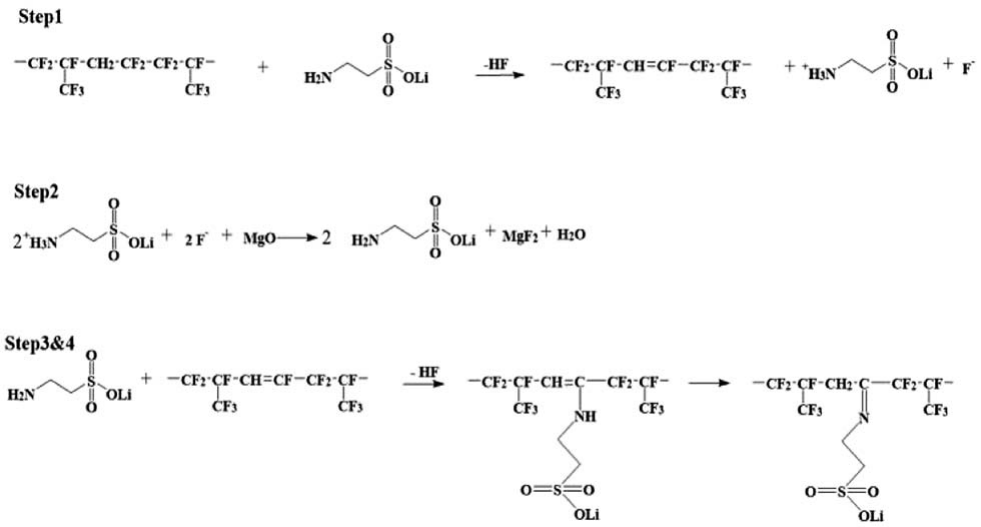
Mechanism of tauric lithium onto P(VDF-HFP) polymer.

As shown in Scheme 1, two steps were used to obtain the lithium sulfonate-grafted P(VDF-HFP) ionomers: first, taurine was transformed to tauric lithium to increase its reactivity with P(VDF-HFP) polymer; second, lithium sulfonate was directly grafted onto P(VDF-HFP) polymer chains. There are four steps for tauric lithium grafted onto P(VDF-HFP) polymer (see Scheme 2). After dehydrofluorination of the HFP-VDF-HFP triad with amine from tauric lithium (Step 1), MgO allows to trap HF and regenerates the amine (tauric lithium Step 2). The formation of water occurs during the reaction. In Step 3, the amine from tauric lithium adds onto the CF=CH double bond through a Michael addition reaction. Finally, the rearrangement leads to the formation of an imine (*i.e.* tauric lithium was grafted onto P(VDF-HFP) polymer).^48^

**Fig. 1:**
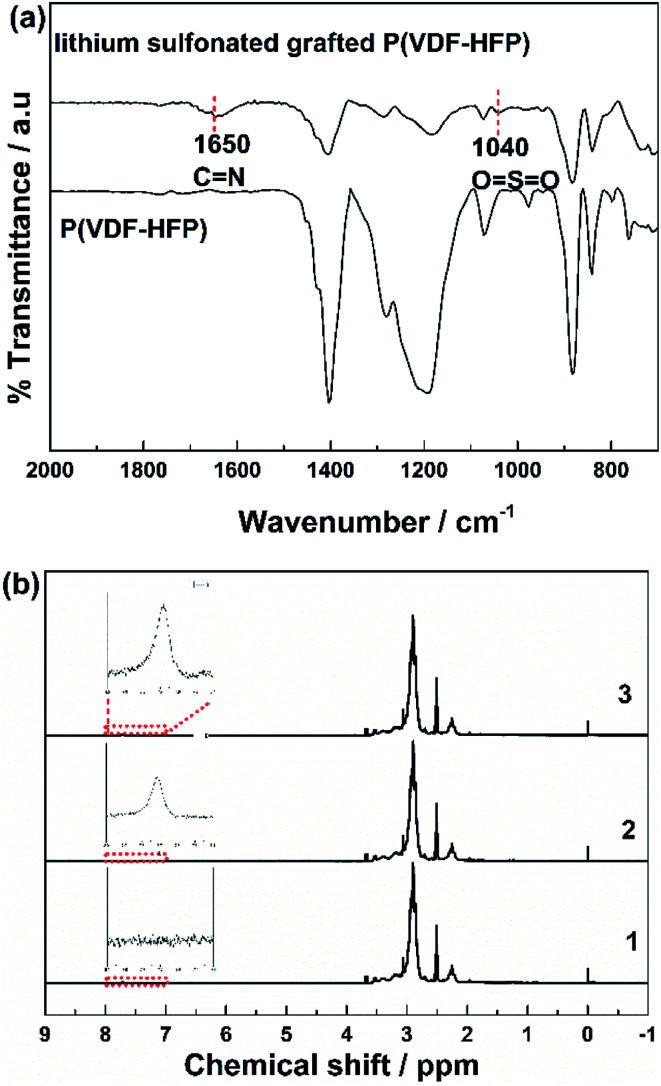
(a) FTIR spectra of P(VDF-HFP) and lithium sulfonate-grafted P(VDF-HFP). (b) ^1^H NMR spectra of P(VDF-HFP) (1) and lithium sulfonate-grafted P(VDF-HFP) with taurine feed content 15 wt% (2) and 20 wt% (3) in DMSO-d_6_, respectively.

Fig. 1a showed FTIR spectra of the P(VDF-HFP) polymer and the lithium sulfonate-grafted P(VDF-HFP) ionomers. It can be found that there was a new characteristic peak at 1635 cm^−1^ attributed to the symmetric stretching vibration of C=N in the lithium sulfonate-grafted ionomers compared to the P(VDF-HFP) polymer.^49^ In addition, the characteristic peak at 1040 cm^−1^ belonging to the symmetric stretching vibration of O=S=O can also be clearly found. It indicated that tauric lithium was successfully grafted onto P(VDF-HFP) polymer chains. The peaks at 1178 and 1400 cm^−1^ were the stretching vibration of CF_2_ and CH_2_, respectively.^50^ It can be found that the intensity of both peaks of CF_2_ and CH_2_ reduced after grafting the tauric lithium.

To further verify this grafted reaction, the lithium sulfonated grafted P(VDF-HFP) was infiltrated in 1 M HCl solution for 72 h to substitute Li^+^ by H^+^. Then, the obtained sulfonic acid-grafted P(VDF-HFP) polymer was tested by ^1^H NMR. Fig. 1b shows ^1^H NMR spectra of P(VDF-HFP) polymer and the sulfonic acid-grafted P(VDF-HFP) with 15 wt% and 20 wt% contents of taurine. The new resonance at 7.7 ppm can be assigned to hydroxyl proton of sulfonic acid group in the sulfonic acid-grafted P(VDF-HFP) compared to the P(VDF-HFP) polymer.^51^ It can also be found that the intensity of this new resonance at 7.7 ppm increased with the increasing of the content of taurine. Both FTIR and ^1^H NMR results demonstrated that tauric lithium was successfully grafted to P(VDF-HFP) polymer in our experiments.

The content of Li^+^ (*i.e.* Li^+^ exchange capacity) in the lithium sulfonate-grafted P(VDF-HFP) ionomers was determined quantitatively by titration method. Table 1 showed the theoretical Li^+^ exchange capacity and the measured Li^+^ exchange capacity. Theoretical Li^+^ exchange capacity of 5 wt%, 15 wt% and 20 wt% taurine feed content were 3.91 × 10^−4^, 1.17 × 10^−3^ and 1.56 × 10^−3^ mmol g^−1^ respectively. Measured Li^+^ exchange capacity of 5 wt%, 15 wt% and 20 wt% taurine feed content were 3.81 × 10^−4^, 1.05 × 10^−3^ and 1.25 × 10^−3^ mmol g^−1^ respectively. The Li^+^ exchange capacity was increased from 0.38 mmol g^−1^ from the ionomer with 5 wt% taurine feed content to 1.25 mmol g^−1^ with 20 wt% taurine feed content.

**Table 1:** Exchange capacity of lithium sulfonated-grafted P(VDF-HFP)

Taurine feed content^a^ (wt%)	Theoretical Li^+^ exchange capacity^b^ (mmol g^−1^)	Measured Li^+^ exchange capacity (mmol g^−1^)
5	3.91 × 10^−4^	3.81 × 10^−4^
15	1.17 × 10^−3^	1.05 × 10^−3^
20	1.56 × 10^−3^	1.25 × 10^−3^

a Calculated by the feed weight ratio of taurine and P(VDF-HFP).

b Assuming 100% conversion.

**Fig. 2:**
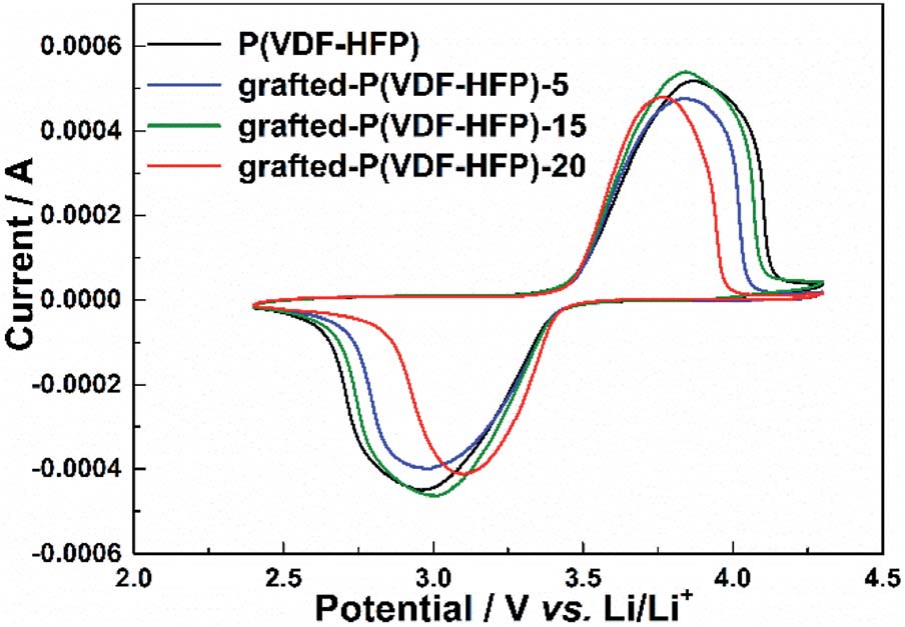
Cyclic voltammograms of the LiFePO_4_ electrodes with P(VDF-HFP) and lithium sulfonate-grafted P(VDF-HFP) binders at the scanning rate of 0.2 mV s^−1^ and room temperature.

The electrochemical stability of the lithium-sulfonate-grafted P(VDF-HFP) binders in the LiFePO_4_ cathode was tested by cyclic voltammetry at room temperature. Fig. 2 showed the CV profiles of the LiFePO_4_ cathodes with the P(VDF-HFP) and the grafted-P(VDF-HFP) binders at a scanning rate of 0.2 mV s^−1^ and room temperature. It can be found that there were a pair of oxidation and reduction peaks corresponding to Fe^2+^/Fe^3+^ redox couple for all the LiFePO_4_ electrodes. Compared to the electrode with P(VDF-HFP) binder, all the electrodes with the grafted-P(VDF-HFP) binders showed lower cathodic potential and higher anodic potential. Furthermore, the electrodes with the grafted-P(VDF-HFP) binders showed a smaller voltage difference between the oxidation and reduction peak potential than that of the electrode with P(VDF-HFP) binder. The voltage differences ranked from P(VDF-HFP) (0.95 V) > grafted-P(VDF-HFP)-5 (0.88 V) > grafted-P(VDF-HFP)-15 (0.83 V) > grafted-P(VDF-HFP)-20 (0.65 V), which indicated that the electrodes with the grafted-P(VDF-HFP) binders will reduce the electrochemical polarization. In addition, the electrode with the grafted-P(VDF-HFP)-20 binder had the lowest electrochemical polarization among all the electrodes.

**Fig. 3:**
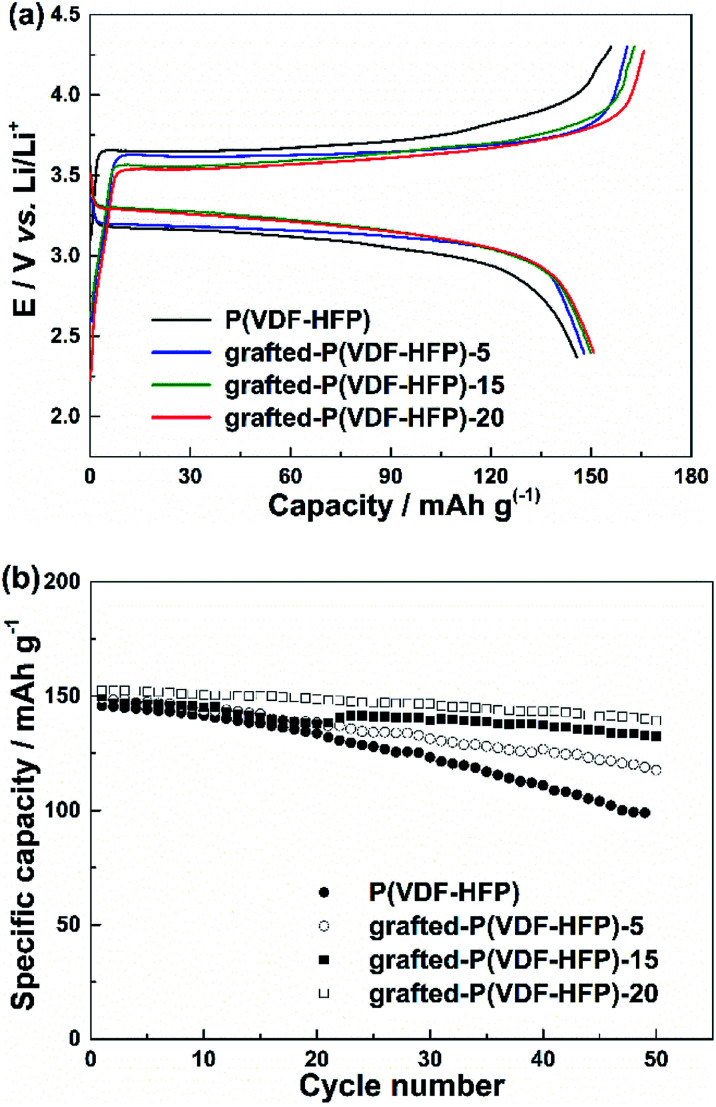
(a) Initial charge and discharge profiles of the LiFePO_4_ electrodes with P(VDF-HFP) and lithium sulfonate-grafted P(VDF-HFP) binders at room temperature and 1C rate. (b) Cycling performance of the LiFePO_4_ electrodes at room temperature and 1C rate.

The cycling performance of the LiFePO_4_ electrodes with the P(VDF-HFP) and grafted-P(VDF-HFP) binders were shown in Fig. 3. The cells were cycled at 1C under constant current conditions at room temperature. Fig. 3a showed the initial charge and discharge curves of the LiFePO_4_ electrodes with different binders. It was clear that all the electrodes with the grafted-P(VDF-HFP) binders showed a higher discharge plateau potential and a lower charge plateau potential than that of the electrodes with the P(VDF-HFP) binder. The similar results have also been reported in other ionomers as binders for LIBs.^13–15^ As shown in Fig. 3b, it was obvious that the cycling performance of the electrodes with the grafted-P(VDF-HFP) binders were better than that of the electrode with the P(VDF-HFP) binder. The capacity fading of the electrode with the P(VDF-HFP) binder was severe, and the discharge specific capacity declined from 145.7 to 99.0 mA h g^−1^ (capacity retention: 67.9%) after 50^th^ cycles. For the electrodes with the grafted-P(VDF-HFP) binders after 50^th^ cycles, the capacity decreased to 118.9 mA h g^−1^ (capacity retention: 79.8%), 132.8 mA h g^−1^ (capacity retention: 89.0%), and 140 mA h g^−1^ (capacity retention: 92.0%) for grafted-P(VDF-HFP)-5 binder, grafted-P(VDF-HFP)-15 binder and grafted-P(VDF-HFP)-20 binder, respectively.

**Fig. 4:**
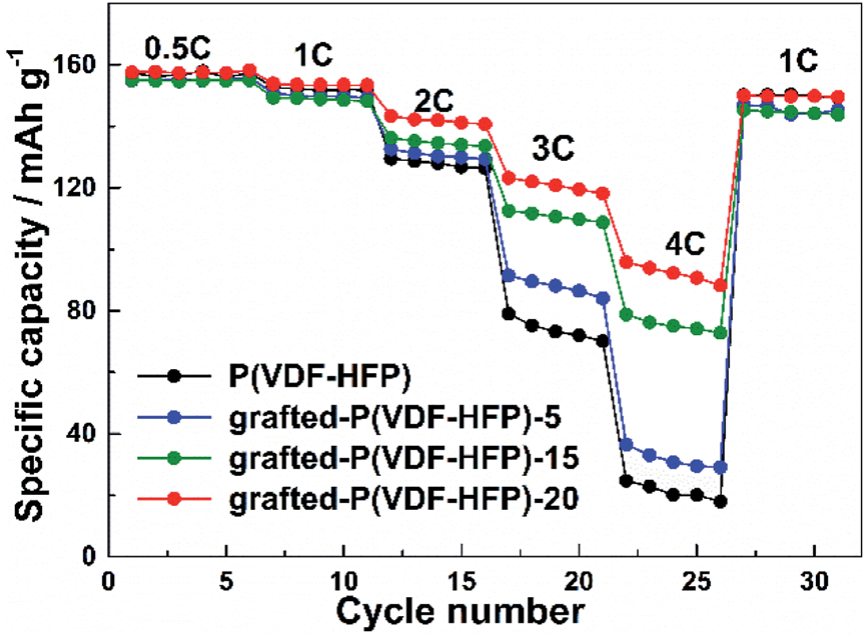
Rate performance of the LiFePO_4_ electrodes with P(VDF-HFP) and lithium sulfonate-grafted P(VDF-HFP) binders.

**Scheme 3:**
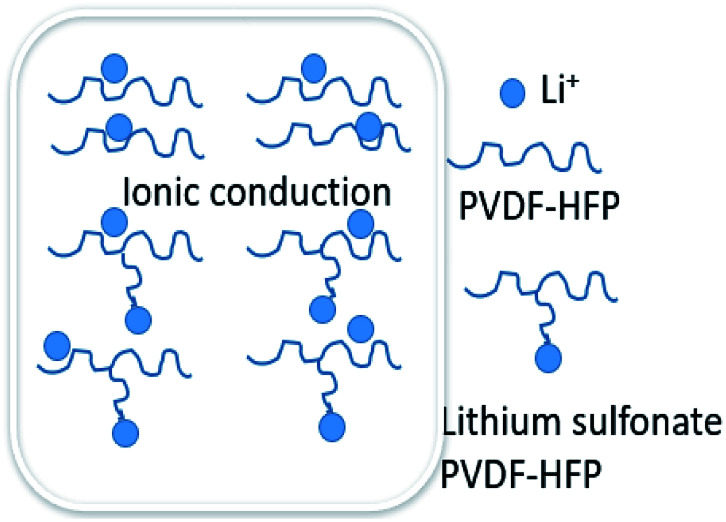
Illustration of a possible mechanism for Li-ion transport in the electrodes with P(VDF-HFP) and lithium sulfonate-grafted P(VDF-HFP) binders.

Fig. 4 showed the rate performance of the electrodes with different binders at various discharge currents ranging from 0.5 C to 4 C. As expected, the discharge specific capacity of all the electrodes gradually decreased with the increasing discharge current density (*i.e.* rate). Compared to the electrode with P(VDF-HFP) binder, however, all the electrodes with the grafted-P(VDF-HFP) binders showed lower decrease in capacity, especially at higher rates. For example, at 4C as shown in Fig. 4, the discharge specific capacity of the electrodes with the grafted binders (grafted-P(VDF-HFP)-5 binder: 36.5 mA h g^−1^, grafted-P(VDF-HFP)-15: 78.8 mA h g^−1^, grafted-P(VDF-HFP)-20: 95.9 mA h g^−1^) were higher than that of the electrode with P(VDF-HFP) binder (24.7 mA h g^−1^). It indicated that the electrodes with the grafted-P(VDF-HFP) binders showed higher rate capability than that of electrode with the P(VDF-HFP) binder. For the grafted binders, the rate performance of the electrodes increases with the increasing content of Li^+^ (*i.e.* Li^+^ exchange capacity) of the lithium sulfonate-grafted P(VDF-HFP) ionomers. And the electrode with the grafted-P(VDF-HFP)-20 binder exhibited the best rate performance among all the electrodes. Due to P(VDF-HFP) binder does not have intrinsic ionic functionality, it will lead to salt concentration polarization and/or salt depletion within the electrodes during high rate charging and discharging, and finally result in the poorer rate capability of the electrodes.^43^ However, the lithium sulfonate-grafted P(VDF-HFP) ionomer can accommodate Li^+^ ions, the loss of Li^+^ ions in the electrolyte due to consumption *via* electrode reactions can be compensated (see Scheme 3).^43^ Therefore, the rate performance of the electrode with the ionomers will be enhanced.

**Fig. 5:**
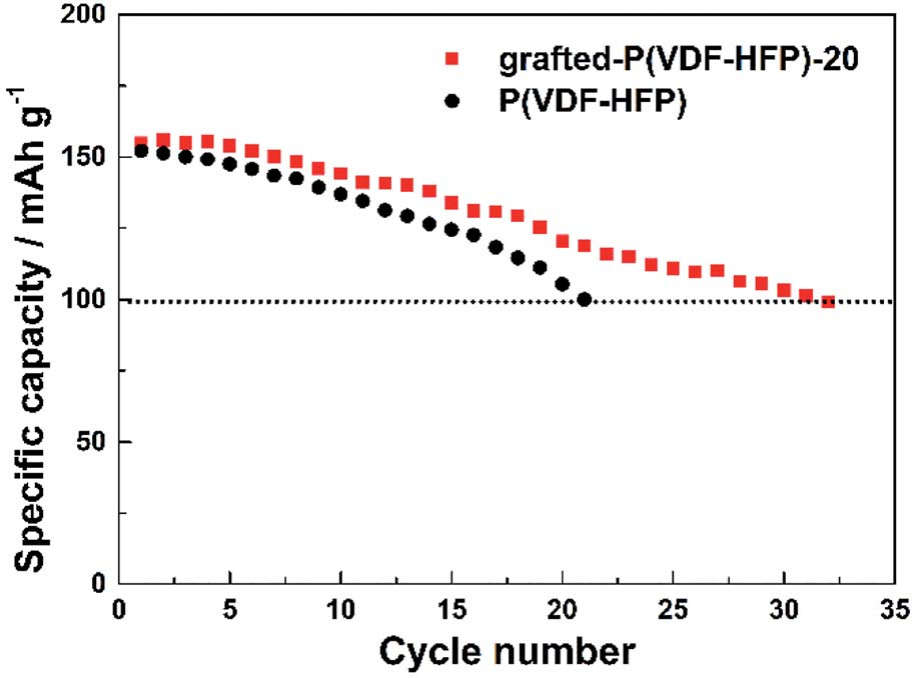
Cycling performance of the LiFePO_4_ electrodes with P(VDF-HFP) and lithium sulfonate-grafted P(VDF-HFP) binders at 60 °C and 1C rate.

**Fig. 6:**
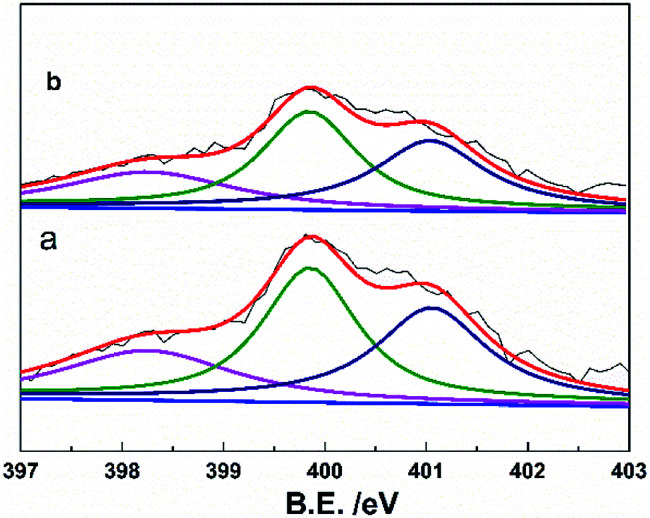
XPS of N1s of the LiFePO_4_ electrodes with lithium sulfonate-grafted P(VDF-HFP) binder before cycling test (a) and (b) after cycling test (32^nd^ cycle) at 60 °C and 1C rate.

Fig. 5 showed the cycling performance of the LiFePO_4_ electrodes at 60 °C and 1C rate. Normally, LiPF_6_-based electrolytes at higher temperature (>50 °C) will result in thermal decomposition of LiPF_6_ to form LiF, PF_5_ and HF, which will lead to not only the decomposition of the electrolyte, but also increase the resistance of LIBs and finally bring about fading of the capacity. In addition, the iron element of LiFePO_4_ will tend towards dissolving in the electrolyte due to the presence of HF. These issues caused that both LIBs with P(VDF-HFP) and grafted-P(VDF-HFP) binders showed poor thermal stability at 60 °C. However, the electrode with the grafted-P(VDF-HFP)-20 binder exhibited better performance compared to the electrode with P(VDF-HFP) binder (as shown in Fig. 5). In addition, XPS measurements was carried out to verify the stability of grafted-P(VDF-HFP) binders after the cycling test at higher temperature. As shown in Fig. 6a, N1s spectra of the LiFePO_4_ electrode with lithium sulfonate-grafted P(VDF-HFP) binder before cycling test can be fitted into three peaks at 398.2, 399.5, 401.0 eV, corresponding to pyridine-like, pyrrole-like and quaternary-like nitrogen, respectively.^52^ Compared to the electrode before cycling test, we can not find any change for the electrode after cycling test (see Fig. 6). These results indicated that the grafted-P(VDF-HFP) binder was stable during the cycling test, even at higher temperature. The reason maybe the C=N double bond in the grafted-P(VDF-HFP) polymer was highly reactive towards nucleophiles due to the polarization by both CF_3_ and CF(CF_3_) perfluoroalkyl groups.

**Fig. 7:**
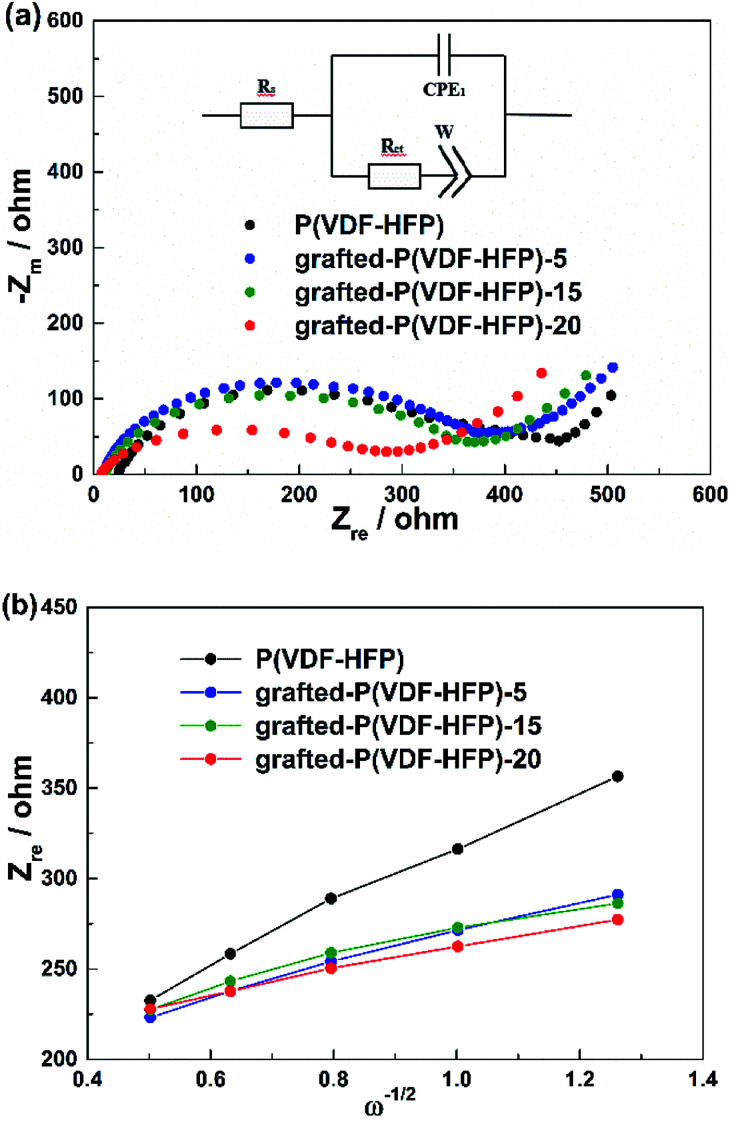
(a) EIS results of the LiFePO_4_ electrodes with P(VDF-HFP) and lithium sulfonate-grafted P(VDF-HFP) binders at frequencies from 0.01 Hz to 100 kHz. (b) Plots of *Z*′ − *ω*^−1/2^ circuit.

Fig. 7a showed the EIS curves of the LiFePO_4_ electrodes with the P(VDF-HFP) and grafted-P(VDF-HFP) binders. An intercept at *Z*_re_-axis in the high frequency corresponds to the ohmic resistance (*R*_s_). The semicircle in the middle frequency range was attributed to the charge-transfer reaction resistance (*R*_ct_) in the cathode-electrolyte interface. The straight line in the lower frequency region represented the Warburg impedance (*W*), which was associated with Li^+^ diffusion in the LiFePO_4_/C cathode. A simplified equivalent circuit was constructed to analyse the impedance spectra in Fig. 7a by ZSimpWin V 3.1 program. The lithium ion diffusion coefficient (*D*_Li_) can be calculated from the formula as following:^53^1
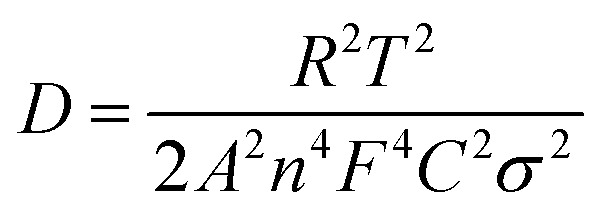

2 *Z*′ = *R*_e_ + *R*_f_ + *R*_ct_ + *σ*_w_*W*^−1/2^

herein *A* is the surface area of the electrode, *F* is the Faraday constant, *C* is the concentration of lithium ion in the electrode, *n* is the number of electrons per molecule during oxidization, *R* is the gas constant, *T* is the room temperature, *σ* is the Warburg factor which can be obtained from the line of *Z*′–*ω*^−1/2^ (shown in Fig. 7b). The calculated lithium diffusion coefficient for the electrodes with different binders were listed in Table 2 according to the above formula. From Table 2, it can be found that the electrodes with the grafted-P(VDF-HFP) binders showed higher lithium diffusion capability (*i.e.* higher *D*_Li_ value) than that of the electrode with P(VDF-HFP) binder. It can be attributed to the lithium sulfonate-grafted P(VDF-HFP) ionomer within the electrode will increase the migration number of Li^+^ during the charge and discharge processes. And the electrode with the grafted-P(VDF-HFP)-20 binder exhibited the highest lithium diffusion capability among all the electrodes.

**Table 2:** Impedance parameters derived using equivalent circuit model and lithium diffusion coefficient for different binders of LiFePO_4_/C cathode

Binders	*R*_s_ (Ω)	*R*_ct_ (Ω)	*D*_Li_ (cm s^−1^)
P(VDF-HFP)	22.52	365.1	1.83 × 10^−14^
Grafted-P(VDF-HFP)-5	9.663	342.0	6.05 × 10^−14^
Grafted-P(VDF-HFP)-15	10.69	329.1	8.11 × 10^−14^
Grafted-P(VDF-HFP)-20	4.43	285.0	1.14 × 10^−13^

The adhesion ability between current collector and active material was a vital factor to choose suitable binder. In our experiments, 180 peeling test was employed to evaluate the adhesion strength between the coating electrode and the Al current. Since the electrode with the grafted-P(VDF-HFP)-20 binder showed the best electrochemical performance among all the electrodes based on the above-mentioned results, we further measured the adhesion ability of this electrode. The peeling strength of the electrodes with the P(VDF-HFP), grafted-P(VDF-HFP)-20 binders and PVDF are 0.96 ± 0.01 N, 1.22 ± 0.01 N and 0.4 N ± 0.01 N, respectively. The result indicated that the adhesion of the electrode with the grafted-P(VDF-HFP)-20 binder was stronger than that of the electrode with the P(VDF-HFP) binder and PVDF.

## Conclusion

In this work, we successfully synthesized the lithium sulfonate-grafted P(VDF-HFP) ionomers with different content of Li^+^ through the attachment of taurine, and used them as binders of the electrodes in LIBs and Li–S batteries. The results have proved that the lithium sulfonate-grafted P(VDF-HFP) ionomers exhibited good electrochemical performance as binders for less resistance SEI film and faster charge transfer during charge–discharge process. The binders containing lithium ionomers increased the available amount of lithium ions in the composite electrodes, resulting in the improvement of rate capability of LIBs. Considering taurine was ubiquitously distributed in animal tissues and can be chemically synthesized at low cost, we believe that the lithium sulfonate-grafted P(VDF-HFP) ionomers will be cheap and available. Therefore, the lithium sulfonate-grafted P(VDF-HFP) ionomers offer a new route to improve electrochemical performance of batteries.

## Conflicts of interest

There are no conflicts to declare.

## Supplementary Material
